# Di-n-Butyl-, Tri-n-Butyl- and Triphenyltin dl-Terebates: Synthesis, Characterization and *In Vitro* Antitumour Activity

**DOI:** 10.1155/MBD.1997.193

**Published:** 1997

**Authors:** Marcel Gielen, Huairang Ma, Abdeslam Bouhdid, Hassan Dalil, Monique Biesemans, Rudolph Willem

**Affiliations:** 1 Department of General and Organic Chemistry Faculty of Applied Sciences Free University of Brussels V.U.B. Pleinlaan 2 Brussels B-1050 Belgium; 2 High Resolution NMR Centre Free University of Brussels V.U.B. Pleinlaan 2 Brussels B-1050 Belgium; 3 Department of Chemistry Northwest University ShaanXi Xian 710 069 China

## Abstract

Di-n-butyltin, tri-n-butyltin and triphenyltin terebates were screened against several human tumour cell lines and found comparably or more active than carboplatin, cis-platin, 5-fluorouracil, methotrexate and doxorubicin, some reference compounds used clinically.


### DI-n-BUTYL-, TRI-n-BUTYL- AND TRIPHENYLTIN dl-TEREBATES: SYNTHESIS, CHARACTERIZATION AND *IN VITRO* ANTITUMOUR ACTIVITY

Marcel Gielen[1a], Huairang Ma[1a,@], Abdeslam Bouhdid[1a], Hassan Dalil[1a], Monique Biesemans[1a, 1b] and Rudolph Willem[1a, 1b]

[1] Free University of Brussels V.U.B., Pleinlaan 2, B-1050 Brussels, Belgium
[a] Department of General and Organic Chemistry, Faculty of Applied Sciences
[b] High Resolution NMR Centre

### Abstract

Di-n-butyltin, tri-n-butyltin and triphenyltin terebates were screened against several human tumour cell lines and found comparably or more active than carboplatin, cis-platin, 5-fluorouracil, methotrexate and doxorubicin, some reference compounds used clinically.

### Introduction

A large number of di- and triorganotin carboxylates exhibit interesting *in vitro* antitumour activities against human tumour cell lines(1).
Several 1:1 and 1:2 condensation compounds of di-n-butyltin oxide with mono-(2a), di-(2b), tri-, tetra-(2c) and pentafluorobenzoic acids(2d) are among such substances. Di-n-butyltin compounds are generally much more active than other diorganotin compounds.
Many substituted triphenyltin benzoates(3) are even more active. In contrast, tri-n-butyltin difluorobenzoates(4) are less active than the corresponding triphenyltin and di-n-butyltin compounds.
A series of tri- and diorganotin steroidcarboxylates were recently screened against seven human tumour cell lines, MCF-7 and EVSA-T, two breast cancers, WiDr, a colon cancer, IGROV, an ovarian cancer, M19 MEL, a melanoma, A498, a renal cancer, and H226, a non small cell lung cancer. The *in vitro* antitumour activities of the di-n-butyltin compound lie between those of 5-fluorouracil and doxorubicin. The activities of the triorganotin compounds are comparable to those of methotrexate or doxorubicin(5).
In the frame of our interest in biologically relevant organotin carboxylates, we report the synthesis and antitumour properties of di- and triorganotin derivatives of dl-terebic acid.

### Results and Discussion

Di-n-butyltin diterebate, $(H_9O_2C_6\text{-}COO)_2SnBu_2$, compound **1**, tri-n-butyltin terebate, $(H_9O_2C_6\text{-}COO)SnBu_3$, compound **2**, and triphenyltin terebate, $(H_9O_2C_6\text{-}COO)SnPh_3$, compound **3**, were synthesized by the condensation of dl-terebic acid, $(H_9O_2C_6\text{-}COOH)$ with respectively di-n-butyltin oxide, tri-n-butyltin acetate and triphenyltin hydroxide.
They were characterized by $^{1}H$ NMR, through multiplet patterns and resonance integrals, by $^{13}C$ NMR, supported by DEPT experiments, by $^{117}Sn$ NMR and by $^{119}Sn$ Mössbauer spectroscopy (see Experimental Section).
The $^{1}J(^{13}C\text{-}^{119/117}Sn)$ coupling constants (567/542 Hz) and the $^{117}Sn$ chemical shift (-131.6 ppm) of compound **1** lie in the ranges characteristic for di-n-butyltin dibenzoates(6) and -disalicylates(7)(8) displaying a pseudo-octahedral or alternatively trapezoidal bipyramidal structure with two n-butyl groups in axial positions. The carboxylates coordinate the tin atom in the bidentate mode in equatorial positions with two adjacent short and two longer Sn-O bonds. All other NMR resonances are, accordingly, as expected for this type of structure.

@ Present address: Department of Chemistry, Northwest University, Xian, ShaanXi 710 069, P. R. China

The $^{1}J(^{13}C-^{119/117}Sn)$ coupling constants (354/338 Hz) and the $^{117}Sn$ chemical shift (+125.4 ppm) of compound **2** are compatible with a monomeric tetrahedral structure found recently for a series of tri-n-butyl fluorobenzoates in solution(4), in agreement with earlier coupling data described by Holecek and Lycka(9).

The $^{1}J(^{13}C-^{119/117}Sn)$ coupling constants (648/618 Hz) and the $^{117}Sn$ chemical shift (-97.6 ppm) of compound **3** are again in agreement with a tetrahedral monomeric structure(2)(10)(11).

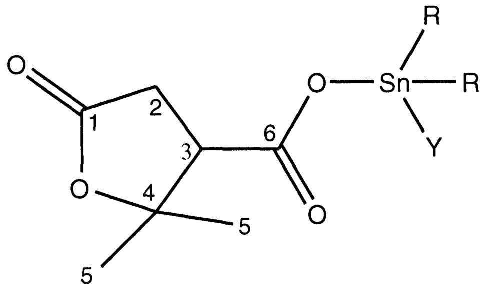

Y =

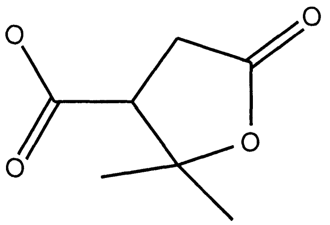

R = $CH_2CH_2CH_2CH_3$, compound **1**
7 8 9 10

R = Y = $CH_2CH_2CH_2CH_3$, compound **2**
7 8 9 10

R = Y =

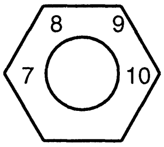

, compound **3**

### Antitumour activities

The *in vitro* antitumour activities of compounds **1**, **2** and **3** against several human cell lines, MCF-7 and EVSA-T, two breast cancers, WiDr, a colon cancer, IGROV, an ovarian cancer, M19 MEL, a melanoma, A498, a renal cancer, and H226, a non small cell lung cancer, are displayed in table 1, together with those of some reference compounds used clinically: carboplatin, cis-platin, 5-fluorouracil, methotrexate and doxorubicin.

Table 1 clearly shows that the compounds are comparably to more active than methotrexate and doxorubicin *in vitro* against almost all cell lines. The very high activity of compounds **2** and **3** against EVSA-T as well as of compound **2** in general should be outlined.

### Experimental part

### *Syntheses*

Compounds **1** to **3** were prepared by adding 5 mmole of di-n-butyltin oxide, tri-n-butyltin acetate or triphenyltin hydroxide, respectively, to a solution of 10 mmole, 5 mmole or 5 mmole of terebic acid in 150 $cm^3$ toluene and 50 $cm^3$ ethanol. After refluxing for 6 h, distilling off the ternary azeotrope water/toluene/ethanol with a Dean Stark funnel and half of the remaining solvent, the resulting mixture was cooled down to room temperature, filtered and evaporated under vacuum. The residue was recrystallized from the appropriate solvent.

| Compounds | MCF-7 | EVSA-T | WiDr | IGROV | M19 MEL | A498 | M226 |
|---|---|---|---|---|---|---|---|
| **1** | 27 | 25 | 134 | 18 | 27 | 61 | 104 |
| **2** | 3 | <3 | 11 | 4 | 11 | 15 | 8 |
| **3** | 17 | <3 | 17 | 19 | 42 | 58 | 39 |
| Carboplatin(14) | 10500 | 4500 | 3500 | 2400 | 5500 | 18000 | 25000 |
| Cisplatin(14) | 1400 | 920 | 1550 | 230 | 780 | 1200 | 3158 |
| 5-Fluorouracil(14) | 350 | 720 | 440 | 850 | 310 | 340 | 5300 |
| Methotrexate(14) | 15 | 26 | 7 | 20 | 18 | 16 | 70 |
| Doxorubicin(14) | 25 | 13 | 18 | 150 | 21 | 55 | 180 |

Table 1. *In vitro* antitumour activities (ng/mL) of compounds **1** to **3**, together with those of some reference compounds used clinically.

*Characterization*

Mössbauer data: QS: quadrupole splitting; IS: isomer shift; $\Gamma_1$ and $\Gamma_2$: line widths, all in mm/s.
NMR data: all spectra were acquired from $CDCl_3$ solutions and referenced to the residual $C^1HCl_3$ resonance at 7.24 ppm for the $^1H$ spectrum, to the central $^{13}CDCl_3$ resonance at 77.0 ppm for the $^{13}C$ spectrum and to $\Xi(^{119}Sn)$ = 37.290665 for the $^{119}Sn$ spectra(12).
Abbreviations for coupling patterns: s = singlet; d = doublet; t = triplet; td = triplet of doublets; tq = triplet of quartets; b = broad resonance; m = complex pattern; nv = non visible; coupling constants are given in Hz between parentheses for $^nJ(^1H-^1H)$ for $^1H$ spectra. Other coupling constants are indicated explicitely.
Compound **1**: recrystallization solvent: chlorofom/n-hexane; m.p.: 98-100°C; yield: 85%; Mössbauer data: QS: 3.44; IS: 1.34; $\Gamma_1$: 0.99; $\Gamma_2$: 0.91; $^1H$ NMR data: 2.988, dd (18, 9): H-2a; 2.700, dd (18, 9): H-2b; 3.196, dd (9, 9): H-3; 1.581, s: H-5a; 1.348, s: H-5b; 1.62 - 1.64, m: H-7 & H-8; 1.25 - 1.40, m: H-9; 0.873, t (7): H-10; $^{13}C$ NMR data: C-1: 174.1; C-2: 32.5; C-3: 50.5; C-4: 84.5; C-5a: 28.5; C-5b: 23.5; C-6: 179.5; C-7: 25.9 [$^1J(^{119/117}Sn-^{13}C)$ = 573/550]; C-8: 26.7 [$^2J(^{119/117}Sn-^{13}C) \approx 31$]; C-9: 26.4 [$^3J(^{119/117}Sn-^{13}C)$ = 100/96]; C-10: 13.5; $^{119}Sn$ NMR data: -134.0.
Compound **2**: recrystallization solvent: petroleum ether; m.p.: 50-51°C; yield: 95%; Mössbauer data: QS: 3.77; IS: 1.47; $\Gamma_1$: 0.85; $\Gamma_2$: 0.86; $^1H$ NMR data: 2.985, dd (17, 10): H-2a; 2.622, dd (17, 8): H-2b; 3.121, dd (10, 8): H-3; 1.562, s: H-5a; 1.297, s: H-5b; 1.50 - 1.65, m: H-7 & H-8; 1.20 - 1.40, m: H-9; 0.878, t (7): H-10; $^{13}C$ NMR data: C-1: 174.4; C-2: 32.7; C-3: 51.4; C-4: 84.8; C-5a: 28.5; C-5b: 23.3; C-6: 174.8; C-7: 16.7 [$^1J(^{119/117}Sn-^{13}C)$ = 356/338]; C-8: 27.8 [$^2J(^{119/117}Sn-^{13}C) \approx 21$]; C-9: 27.0 [$^3J(^{119/117}Sn-^{13}C) \approx 64$]; C-10: 13.6; $^{119}Sn$ NMR data: 123.4.
Compound **3**: recrystallization solvent: ethanol/petroleum ether; m.p.: 125-126°C; yield: 95%; Mössbauer data: QS: 3.52; IS: 1.31; $\Gamma_1$: 0.82; $\Gamma_2$: 0.79; $^1H$ NMR data: 3.065, dd (18, 10): H-2a; 2.669, dd (18, 9): H-2b; 3.231, dd (10, 9): H-3; 1.585, s: H-5a; 1.114, s: H-5b; 7.70-7.80, m: H-8; 7.30-7.50, m: H-9 & H-10; $^{13}C$ NMR data: C-1: 174.5; C-2: 32.7; C-3: 50.8; C-4: 84.8; C-5a: 28.5; C-5b: 23.2; C-6: 175.6; C-7: 137.5 [$^1J(^{119/117}Sn-^{13}C)$ = 648/618]; C-8: 136.9 [$^2J(^{119/117}Sn-^{13}C)$ = 51/49]; C-9: 129.1 [$^3J(^{119/117}Sn-^{13}C)$ = 65/62]; C-10: 130.5 [$^4J(^{119/117}Sn-^{13}C) \approx 13$]; $^{119}Sn$ NMR data: -98.3.

*Instruments*

All NMR spectra were recorded on a Bruker AC250 instrument, using a QNP probe tuned at 250.13, 62.93 and 93.28 MHz for $^1H$, $^{13}C$ and $^{119}Sn$ nuclei, respectively.
Mössbauer spectra were obtained as described previously(8).

*In vitro tests*

The following human tumor cell lines were used: - MCF7 Breast cancer - EVSA-T Breast cancer - WiDr Colon cancer - IGROV Ovarian cancer - M19 MEL Melanoma - A498 Renal cancer - H226 Non small cell lung cancer.

MCF7 is estrogen receptor ER+ /progesterone receptor PgR+ and EVSA-T is ER-/PgR-.
Cell lines WIDR, M19 MEL, A498, IGROV and H226 belong to the currently used anti-cancer screening panel of the National Cancer Institute, USA(13). The *in vitro* cytotoxicity of the compounds was determined using SRB(14) as a cell viability test.
Prior to the experiments a mycoplasma test was carried out on all cell lines and found to be negative. All cell lines were maintained in a continuous logarithmic culture in RPMI 1640 medium with Hepes and phenol red. The medium was supplemented with 10% FCS, penicillin 100 IU/ml:md streptomycin 100 μg/ml. The cells were mildly trypsinized for passage and for use in experiments. RPMI and FCS were obtained from Life technologies (Paisley, Scotland). SRB, DMSO, penicllin and streptomycin were obtained from Sigma (St. Louis, MO, USA), TCA and acetic acid from Baker B.V. (Deventer, NL) and PBS from NPBI B.V. (Emmer-Compascuum, NL).
The test and reference compounds were dissolved to a concentration of 238095 ng/ml in full medium, by 21 fold dilution of a DMSO solution which contained 1 mg compound / 200 μl . The experiment was started on day 0.
On day 0 150 μl of trypsinized tumor cells (1500 - 2000 cells/well) were plated in 96-wells flatbottom microtiter plates (falcon 3072, BD). The plates were preincubated 48 hr at 37°C, 8,5 % $CO_2$ to allow the cells to adhere.
On day 2, a three fold dilution sequence of ten steps was made in full medium, starting with the 238095 ng/ml stock solution. Every dilution was used in quadruplicate by adding 50 μl to a column of four wells.
This results in a highest concentration of 59523 ng/ml present in column 12. Column 2 was used for the blank. To column 1 PBS was added to diminish interfering evaporation.
On day 7 the incubation was terrninated by washing the plate twice with PBS. Subsequently the cells were fixed with 10% trichloroacetic acid in PBS and placed at 4°C for one hour. After five washings with tap water, the cells were stained for at least 15 minutes with 0,4% SRB dissolved in 1% acetic acid. After staining the cells were washed with 1% acetic acid to remove the unbound stain. The plates were air dried and the bound stain was dissolved in 150 μl tris base. The absorbance was read at 540 nm using an automated microplate reader (Labsystems Multiskan MS). Data were used for construction of concentration-response curves and determination of the $ID_{50}$ value by use of Deltasoft 3 software.
All raw data and the mastercopy of this report have been filed in the archives of the laboratory of medical oncology of the Rotterdam Academical hospital.

### Acknowledgements

We thank Mrs. I. Verbruggen for the acquisition of the NMR data and Dr. B. Mahieu for the acquisition of the Mössbauer spectra. We are indebted to Dr. D. de Vos, Mr. H. J. Kolker, Dr. J. Verweij, Prof. Dr. G. Stoter, Dr. J. H. M. Schellens for the *in vitro* tests. This research was supported by the Belgian "Nationaal Fonds voor Wetenschappelijk Onderzoek" N.F.W.O. (grant number G.0054.96, M.G.), by the Belgian "Nationale Loterij" (grant number 9.0006.93, R.W., M.B.), the Belgian "Fonds voor Kollektief Fundamenteel Onderzoek" (grant number 9.0094.94, R.W., M.B.) and by the Human Capital and Mobility Programme of the European Community (Contract Nr ERBCHRXCT92-0016).

### References

(1) see for instance Gielen, M.; Lelieveld, P.; de Vos, D.; Willem, R. in "Metal Complexes in Cancer Chemotherapy", Ed.: Keppler, B.K.; VCH, Weinheim, **1993**, chapter 17, p. 383; Gielen, M., *Coord. Chem. Rev.*, **1996**, *151* , 41; Gielen, M., in "Cytotoxic, mutagenic and carcinogenic potential of heavy metals related to human environment", NATO ASI Series 2: Environment - Vol. 26, Ed.: Hadjiliadis, N. D., Kluwer Academic Publishers, **1997**, pp. 445 - 455

(2) (a) Gielen, M.; El Khloufi, A.; Biesemans, M.; Willem, R; *Appl. Organomet. Chem.*, **1992**, *7*, 119; (b) Gielen, M.; El Khloufi, A.; Biesemans, M.; Kayser, F.; Willem, R.; *Appl. Organomet. Chem.* ,**1993**, *7* , 201; (c) Gielen, M.; Biesemans, M.; El Khloufi, A.; Meunier-Piret, J.; Kayser, F.; Willem, R.; *J. Fluorine Chem.*, **1993**, *64*, 279; (d) Gielen, M.; El Khloufi, A.; de Vos, D.; Kolker, H. J.; Schellens, J. H. M.; Willem, R., *Bull. Soc. Chim. Belg.*, **1993**, *102*, 761; (e) Willem, R.; Bouhdid, A.; Biesemans, M.; Martins, J. C.; de Vos, D.; Tiekink, E. R. T.; Gielen, M., *J. Organomet. Chem.*, **1996**, *514* , 203

(3) Gielen, M.; Willem, R.; Biesemans, M.; Bouâlam, M.; El Khloufi, A.; de Vos, D., *Appl. Organomet. Chem.*, **1992**, *6*, 287; Gielen, M.; El Khloufi, A.; Biesemans, M.; Bouhdid, A.; de Vos, D.; Mahieu, B.; Willem, R., *Metal-Based Drugs*, **1994**, *1*, 305

(4) Gielen, M.; El Khloufi, A.; Biesemans, M.; Kayser, F.; Willem, R.; Mahieu, B.; Maes, D.; Lisgarten, J. N.; Wyns, L.; Moreira, A.; Chattopadhay, T. K.; Palmer, R. A., *Organometallics*, **1994**, *13*, 2849
(5) Willem, R.; Dalil, H.; Broekaert, P.; Biesemans, M.; Ghys, L. , Nooter, K.; de Vos, D.; Gielen, M., *Main Group Met. Chem.*, in press
(6) Gielen, M.; El Khloufi, A.; Biesemans, M.; Mahieu B.; Willem, R., *Bull. Soc. Chim. Belg.* **1992**, *101* , 243; Gielen, M.; Meunier-Piret, J.; Biesemans, M.; Willem, R.; El Khloufi, A., *Appl. Organomet. Chem.*, **1992**, *6*, 59; Gielen, M.; Meunier-Piret, J.; Biesemans, M.; El Khloufi A.; Willem, R. *Polyhedron*, **1992**, *11*, 1861; Gielen, M.; El Khloufi, A.; Biesemans M.; Willem, R. *Appl. Organomet. Chem.* ,**1993**, *7*, 119; Gielen, M.; El Khloufi, A.; Biesemans, M.; Kayser F.; Willem, R. *Appl. Organomet. Chem.*, **1993**, *7*, 201; Gielen, M.; Biesemans, M.; El Khloufi, A.; Meunier-Piret J.; Willem, R., *J. Fluorine Chem.*, **1993**, *64*, 279
(7) Bouâlam, M.; Willem, R.; Biesemans M.; Gielen, M., *Appl. Organomet. Chem.* **1991**, *5*, 497
(8) Bouâlam, M.; Willem, R.; Biesemans, M.; Mahieu, B.; Meunier-Piret, J.; Gielen, M., *Main Group Met. Chem.*, **1991**, *14*, 41
(9) Holecek, J.; Nadvornik, M.; Handlir, K.; Lycka, A. *J. Organomet. Chem.*, **1983**, *241*, 177; Nadvornik, M.; Holecek, J.; Handlir, K.; Lycka, A. *J. Organomet. Chem.*, **1984**, *275,* 43; Sandhu, G. K.; Kaur, G.; Holecek, J.; Lycka, A. *J. Organomet. Chem.*, **1989**, *365*, 215
(10) Gielen, M.; El Khloufi, A.; Biesemans, M.; Bouhdid, A.; de Vos, D.; Mahieu, B.; Willem, R. *Metal-Based Drugs* ,**1994**, *1*, 305
(11) Lycka, A.; Nadvornik, M.; Handlir, K.; Holecek, J., *Coll. Czech. Chem. Commun.*, **1984**, *49* , 2903
(12) Mason, J. Multinuclear NMR, Plenum Press, New York, **1987**, p. 627
(13) Boyd, M. R., Status of the NCI preclinical antitumor drug discovery screen. Principles and practice of oncology, **1989**, *3*,1
(14) Skehan, P.; Storeng, R.; Scudiero, D.; Monks, A.; McMahon, J.; Vistica, D.; Warren, J. T.; Bokesch, H.; Kenney, S.; Boyd, M. R., *J. Natl. Cancer Inst.*,**1990**, *82* , 1107; Kepers, Y. P.; Peters, G. J.; Van Ark-Otte, J.; Winograd, B.; Pinedo, H. M., *Eur. J. Cancer*, **1991**, *27*, 897

**Received: April 25, 1997 - Accepted: May 6, 1997 -**
**Received in revised camera-ready format: May 13, 1997**

